# Community voices on factors influencing COVID-19 concerns and health decisions among racial and ethnic minorities in the school setting

**DOI:** 10.3389/fpubh.2022.1002209

**Published:** 2022-10-19

**Authors:** Tara Kenworthy, Sherelle L. Harmon, Agenia Delouche, Nahel Abugattas, Hannah Zwiebel, Jonathan Martinez, Katheryn C. Sauvigné, C. Mindy Nelson, Viviana E. Horigian, Lisa Gwynn, Elizabeth R. Pulgaron

**Affiliations:** University of Miami Miller School of Medicine, Miami, FL, United States

**Keywords:** COVID-19, qualitative, vaccination, health decisions, racial/ethnic minority, school

## Abstract

Racial and ethnic minority communities have been disproportionately affected by COVID-19, but the uptake of COVID-19 mitigation strategies like vaccination and testing have been slower in these populations. With the continued spread of COVID-19 while in-person learning is a priority, school-aged youth and their caregivers must make health-related decisions daily to ensure health at school. It is critical to understand factors associated with COVID-related health decisions such as vaccination, testing, and other health behaviors (e.g., wearing masks, hand washing). Community-engaged campaigns are necessary to overcome barriers to these health behaviors and promote health equity. The aim of this study was to examine COVID-19-related concerns and influences on health decisions in middle and high schools serving primarily racial and ethnic minority, low-income families. Seven focus groups were conducted with school staff, parents, and students (aged 16 years and older). Qualitative data were analyzed using a general inductive approach. Factors related to COVID-19 concerns and health decisions centered on (1) vaccine hesitancy, (2) testing hesitancy, (3) developmental stage (i.e., ability to engage in health behaviors based on developmental factors like age), (4) cultural and family traditions and beliefs, (5) compatibility of policies and places with recommended health behaviors, (6) reliability of information, and (7) perceived risk. We explore sub-themes in further detail. It is important to understand the community's level of concern and identify factors that influence COVID-19 medical decision making to better address disparities in COVID-19 testing and vaccination uptake.

## Introduction

The Coronavirus disease 2019 (COVID-19) pandemic has had a devastating effect on health and wellbeing in the United States. As of July 21, 2022, there were 90.20 million cumulative confirmed cases and 1.03 million deaths from COVID-19 (1, 2). The pandemic has disproportionately affected racial and ethnic minorities of American Indian or Alaska Native, Black or African American, and Hispanic/Latino origins, including children, in terms of infections, hospitalizations, and death (3, 4). Differences in health outcomes by race and ethnicity are related to increased rates of comorbid health conditions (5–7) and social determinants of health, including healthcare access, educational attainment, social contexts, and economic stability (8). For instance, racial and ethnic minorities are more likely to face economic hardships associated with being uninsured and are more likely to have “essential” jobs where risk of infection due to exposure to COVID-19 is increased (6, 7, 9, 10). These interconnected health and social factors illustrate the multiple, interacting systems that impact overall wellbeing of individuals and communities in the context of COVID-19 and call for nuanced investigations of these layered influences on health behaviors and outcomes.

Despite poorer health outcomes of COVID-19, racial and ethnic minority communities have lagged in rates of vaccination and testing (11, 12). These factors were partially attributed to limited access to vaccines and testing early in the pandemic, which have improved as vaccines have been approved for all individuals over the age of 6 months and tests have become widely available throughout the United States for free or low cost (13, 14). The demographic profile of vaccinated individuals has changed over time as well, with Hispanic individuals more likely to receive a vaccine than White counterparts (15). The CDC does not report race/ethnicity data on vaccinations in children, but vaccination rates remain low. As of July 13, 2022, only 59% of 12–17-year-olds, 30% of 5-11-year-olds, and 3% of 6 months-4-year-olds received the two-dose vaccine series for COVID-19 (16). Data on race and ethnicity with regards to testing is more limited, though lower rates of testing have been reported for racial and ethnic minorities, despite increased likelihood of positive test results in both adults and children (17, 18). Other cognitive and social factors such as vaccine hesitancy, cost of healthcare, lack of insurance, and high utilization of emergency rooms may persist for many individuals despite improvements in access (12, 19). There remains a need to understand ongoing disparities and concerns regarding the utilization of strategies to promote health and prevent COVID-19 infection and spread, especially in communities at higher risk of COVID-19 morbidity and mortality.

Several top-down evidence-informed strategies have been attempted for promoting COVID-19 vaccine uptake, including offering incentives, mandates, or contingencies that allow access only for vaccinated individuals (20). While these approaches can be effective, complementary or alternative strategies may be warranted, especially given the persistence of medical mistrust rooted in historical and ongoing social inequality (21). Organizations such as the American Psychological Association (APA) and the CDC emphasize the importance of trust building and community engagement to increase vaccine confidence (22, 23). Furthermore, a meta-analysis of 131 public health interventions targeting individuals of lower socioeconomic status and/or those identifying as a racial/ethnic minority demonstrated that community engagement was effective for improving health outcomes across many conditions (24). Therefore, the current study applies community based participatory research (CBPR), a method of community engagement that “de-center(s) research “expertise” [(25), p. 3]” by viewing community members as knowledgeable experts and involving them in every aspect of the project. Knowledge from researchers and the community is combined to create social change, improve community health, and eliminate health disparities (25).

Within the CBPR framework, qualitative methods are used to identify priorities of key community stakeholders, combined with the external perspective of researchers (26). This methodological approach emphasizes how individuals, themselves, understand and interpret their life experiences and human interactions (27, 28) and is helpful for clarifying the values of community members in their own voice, a perspective often overlooked (29). Qualitative research has been instrumental in complementing epidemiological investigations of health emergencies, such as the 2013–2016 Ebola outbreak (30). It has been crucial in these cases for understanding how or why individuals engage in health behaviors, developing culturally-informed interventions, and evaluating intervention effectiveness (31). Similarly, there is great potential for the contribution of qualitative studies to understanding health behaviors and generating solutions in response to the COVID-19 pandemic (32).

The aim of this study was to use a CBPR approach supported with qualitative methods to engage youth, caregivers, and school staff in focus group discussions about their current concerns related to COVID-19, vaccination, and testing. Ultimately, we aim to use these qualitative data in combination with quantitative survey data (not described here) to inform a school-based COVID-19 health education initiative that addresses the concerns of the school community. This study took place in a racially, ethnically, and linguistically diverse, low-income, urban setting.

## Materials and methods

### Setting

The National Institutes of Health (NIH) Rapid Acceleration of Diagnostics Underserved Populations (RADx-UP) Return to School Initiative provided funding to 16 research teams across the United States, its territories, and Tribal Nations to understand and improve health outcomes as children return to school in the era of COVID-19. The Return to School Initiative is a subset of over 125 RADx-UP projects studying COVID-19 testing patterns in different community settings, with a focus on reducing health disparities in communities most affected by the pandemic. This paper describes findings from one research team's study.

The research team has a long-standing partnership with the local school district, united with the goal of providing primary health care delivery through a publicly funded initiative. The partnership supporting the School Health Initiative (SHI) has maintained school-based health clinics at nine urban, Title 1 public schools in the Southeastern United States for over 20 years. Clinics offer services including medical care, mental/behavioral health care, health screenings such as dental, vision, hearing and obesity, immunizations, health education/promotion, substance abuse counseling, case management, reproductive health services, and other prevailing health problems in children and families. This existing clinical relationship facilitated collaboration with the school district for research purposes.

This study was conducted at two high schools and one middle school that house SHI clinics. These schools are comprised of primarily racial/ethnic minority students from low-income households. During the 2020–2021 school year, at High School A, 83% of students were Black, 13.8% were Hispanic, and 91.0% were eligible for free or reduced lunch; at High School B, 36.1% of students were Black, 61.5% were Hispanic, and 89.4% were eligible for free or reduced lunch; at the Middle School, 71.5% of students were Black, 23.0% were Hispanic, and 89.6% were eligible for free or reduced lunch (33). Many families in this community are recent immigrants from Haiti and South or Central American countries.

### Participants

Participants were included if they were staff, students over the age of 16, or the parent/guardian (described as “caregiver” in the remaining text) of a student at one of the three target schools and able to speak, read and write English, Spanish, or Haitian Creole at a minimum of 5th grade level. A total of 52 participants took part in one of seven focus groups. More participants came from High School B (44.2%) than the other two schools (25.0% from High School A; 30.7% from Middle School). Three focus groups consisted of staff only; two consisted of caregivers only; two consisted of caregivers and students. Because parent/guardian consent was required for student participation, we offered these mixed-role (caregiver and student) focus groups for participant convenience. Most participants were school staff (48.1%), followed by caregivers (32.7%), students (17.3%), and one participant who identified as both a staff member and parent (1.9%; for the purposes of categorizing responses by role, that participant was included in the staff category because that was the context of the focus group in which they participated). Six groups were held in English and one in Spanish. Though some participants spoke Haitian Creole at home, they also reported fluency in English and were able to participate in the English focus groups. The majority of participants were women (71.7%); Hispanic, Latino, or Spanish (51.2%); and Black/African American (50.0%). They ranged from age 16–72 (mean age = 43.9, SD = 15.4), with the majority ages 36–55 years (50.0%). Average annual household income was widely variable between participants, spanning from <$15,000 to over $100,000. Over half of the respondents had annual household incomes of <$50,000. Demographics are described further in Table 1.

**Table 1:** Demographics.

Variable (*n* responses^*^)	**Characteristics**	**Frequency**	**Percent**
School (*n* = 52)	High school A	13	25.0%
	High school B	23	44.2%
	Middle school	16	30.7%
Role (*n* = 52)	School staff	25	48.1%
	Caregiver	17	32.7%
	School staff & caregiver	1	1.9%
	Student	9	17.3%
Age (*n* = 44)	16–18	6	13.6%
	19–35	4	9.1%
	36–55	22	50.0%
	56+	12	27.3%
Race (*n* = 42)	American Indian or Alaska native	1	42.3%
	Black or African American	21	50.0%
	White	13	30.9%
	Some other race	2	4.8%
	More than one race	2	4.8%
	Prefer not to answer	3	7.1%
Ethnicity (*n* = 43)	Not of Hispanic, Latino, or Spanish origin	16	37.2%
	Yes, of Hispanic, Latino, or Spanish origin	22	51.2%
	Prefer not to answer	5	11.6%
Gender (*n* = 46)	Woman	33	71.7%
	Man	12	26.1%
	Prefer not to answer	1	2.2%
Level of education (*n* = 44)	Less than high school	7	15.9%
	High school graduate or GED	2	4.5%
	Some college/technical/vocational degree	9	20.5%
	Bachelor's degree	10	22.7%
	Other advanced degree	14	31.8%
	Prefer not to answer/don't know	2	4.5%
Language spoken at home (*n* = 44)	English only	15	34.1%
	Language other than English:	29	65.9%
	- Spanish - Haitian creole - Haitian creole & French - Other	−20 - 4 - 1 - 4	-69.0% -13.8% -3.4% -13.8%
Total household income 2019 (*n* = 47)	<$15,000	6	12.8%
	$15,000–$24,999	9	19.1%
	$25,000–$49,999	9	19.1%
	$50,000–$99,999	11	23.4%
	$100,000 and above	4	8.5%
	Prefer not to answer	8	17.0%

* There were a total of 52 participants in focus groups. N is indicated for each demographic variable to demonstrate where missing data was present (i.e., *n* < 52). Percentages are calculated based on the sample of respondents for each variable.

### Procedures

This study was approved by the University's Institutional Review Board and the school district's Office of Research. Each school was assigned at least one school champion, who was a school staff member (e.g., teacher, administrator, counselor) who helped facilitate recruitment and outreach efforts between the university and school community and offered their community-based expertise on accuracy of interpretation of data and acceptability of intervention strategies. With the support of each school champion, participants were recruited via study flyers distributed online and throughout schools, announcements made, various school events such as orientations and open houses, school messaging systems, and word of mouth. This was a convenience sample of participants who responded to recruitment efforts; focus groups were stratified by school and participant type (i.e., student, caregiver, or school staff) to promote inclusion of diverse perspectives.

All adult participants were required to complete consent, and children under 18 required assent and parental consent. Prior to participating in the focus group, all participants were administered measures for the broader research study, which required a separate consent form. These measures included basic demographic questions [e.g., gender, age, race, ethnicity, language(s) spoken, education, household income] described here and additional measures of COVID-19-related experiences that are outside the scope of this paper. Participants received $15 gift cards for completing measures and an additional $25 gift card for participating in a focus group.

The seven focus groups were conducted from September through November 2021 and took ~45 min to 1 h each. Focus groups were held on school grounds in a variety of settings, including the teachers' lounge, classroom, media center, and the school-based clinic. They were conducted by one of three doctoral-level research staff. A research staff member bilingual in Spanish facilitated the groups at High School B, which had a high percentage of Spanish-speakers and included one group conducted entirely in Spanish. At least one other staff member was present during each focus group to facilitate consent, take notes, and distribute payment.

Focus groups followed a semi-structured discussion guide (see Supplementary Materials). The discussion guide was developed by research staff to reflect questions about current concerns about COVID-19, hesitations about vaccination, and hesitations about testing. Additional questions explored how to implement related health education programs, which will be reported in a separate manuscript. The questions were designed to elicit participants' culturally informed conceptualization of problems and solutions, inspired in part by the Cultural Formulation Interview used in psychological assessment settings (34).

The audio from each group was recorded to allow for transcription. Focus groups were transcribed verbatim by study staff. The one focus group conducted in Spanish was transcribed and translated to English by a bilingual member of study staff.

### Qualitative analysis

Qualitative data were analyzed using a general inductive approach, which involves the reading of data to derive themes or models (35). Two doctoral-level team members (TK and SH) closely reviewed all transcripts and collaboratively identified data-derived preliminary themes. Themes were aggregated and defined in a codebook. Four additional coders were then trained on the preliminary codebook. Transcripts were then assigned to coding pairs. Coders independently coded assigned transcripts, then reviewed codes with their partner. Following initial coding, the study team met to review themes and discuss comments and common discrepancies with the coding team. The codebook was refined based on this discussion. All interviews were then re-coded with the updated codebook. Coding pairs reached 100% consensus on all codes.

Initial coding was conducted using the comments feature on a word processor. Once consensus was reached, transcripts and codes were then transferred to NVivo (36) for qualitative analysis. NVivo was used to extract quotes for each code and explore themes using various queries. Specifically, we examined themes by participant role (staff, caregiver, student). Using NVivo's matrix coding query feature, we compared how many participants across roles mentioned each theme. This practice of dealing quantitatively with qualitative data by counting frequencies was conducted in an exploratory manner following the determination of themes using the process previously described. Some may consider frequencies a proxy for significance, but there are limitations including the threat of removing concepts from their context or concepts occurring more frequently due to reasons outside of significant meaning [e.g., a person's greater willingness to discuss, interviewer interest and probing (37)].

To ensure trustworthiness of data, a construct check was conducted for text excerpts, ensuring that they are consistent with construct definitions (38). Edits were made as needed by a coding pair who then reached consensus. Additionally, member checking, which involves sharing a summary of findings with stakeholders (39, 40), is inherent in CBPR because community members are involved throughout the research process (26). This was accomplished through (1) reflecting on emerging themes throughout the focus groups by summarizing participant responses in real time and checking for accurate interpretation and (2) following collection of all focus group data and qualitative analysis (as described previously), solicitation of feedback from school champions on accuracy and completeness of themes.

## Results

Factors underlying COVID-19-related concerns and health decisions were organized within seven broad themes: (1) vaccine hesitancy, (2) testing hesitancy, (3) developmental stage, (4) cultural and family traditions and beliefs, (5) compatibility of policies and places with recommended health behaviors, and (6) reliability of information. These factors were related to (7) perceived risks, which ranged from apathy, to concerns impacting thoughts, and concerns impacting health behaviors (e.g., vaccination, masking, etc.) related to COVID-19. Each theme was further categorized into subcategories related to specific concerns and factors. See Table 2 for definitions of each theme and subtheme and illustrative quotes, which correspond to the numbers in parentheses in the text below. See Table 3 for frequencies of participants endorsing each theme by role (i.e., staff, student, or caregiver).

**Table 2:** Factors related to COVID-19 concerns and health behaviors.

**Theme**	**Definition and Sub-themes**	**Illustrative Quotes**
1. Vaccine Hesitancy	Concerns about COVID-19 vaccines related to: a) *Potential side effects—*health problems that may occur as a result of the vaccine b) *Vaccine development—*the speed of development, rollout of the vaccine, and/or desire for more evidence of safety c) *Content of vaccine—*potential harm of components of the vaccine d) *Vaccine effectiveness—*uncertainty about how well the vaccine will protect against infection or hospitalization e) *Age of recipient—*influence of age on decision to vaccinate	*1a*. “And then some people get a reaction, they die... Eventually there are 20,000 positive things happening, you see the negative, you focus on that. When we took the Johnson (& Johnson), remember? Eight women get affected, some of them died...”—**High School A Staff** *1b*. “I think a lot of people are on the fence because it's new, so there's not a lot of data, there's not a lot of research about it, so you're saying, ‘Put this in your body. We think it helps but we don't know what else it does.” - **Middle School Staff** *1c*. “I've been asking myself, like, what type of substance do they use for them to make the vaccine?... But for me, I'm allergic to chocolate and those things. Imagine they but those things in some of the chemicals in there...”—**High School A Student** *1d*. “They were saying initially that if you got vaccinated, you're not going to get COVID. They didn't say that, you know, even though you may have got vaccinated, there is a possibility that you could still contract COVID and spread it to your family, like my mom did (laughs).”—**Middle School Caregiver** *1e*. “Well, I have three kids, 15, 12, and 7... I got vaccinated because I'm the mother and father for them. I am the one who works and maintains my home, but I prefer not to vaccinate my children.”—**High School B Caregiver**
2. Testing Hesitancy	Concerns related to COVID-19 testing procedure or results including: a) *Reliability of testing—*possibility of unreliable test results (e.g., false positives) b) *Consequences of positive test—*negative consequences following COVID-19 diagnosis	*2a*. “I have very bad sinus infections...and at the times when I'm going through having a sinus infection, I will not do a COVID test... I'm afraid that I'll go in and because I have a stuffy nose or something, they're gonna say, ‘Oh, you have COVID.”'—**Middle School Caregiver** *2b*. “Especially if they're athletes, they're hesitant to get tested because they know that means they won't practice.” - **High School B Staff**
3. Developmental Stage	Perceived influence of developmental stage or age on health behaviors	*3a*. “As adults say, we, the children, grab everything... and we don't want to put on the mask because we can't breathe...”—**High School B Student**
4. Cultural and family traditions and beliefs	Health beliefs and/or behaviors influenced by family or cultural beliefs or practices, specifically: a. *Family practices*—health beliefs/behaviors influenced by family culture b. *Cultural practices*—health beliefs/behaviors influenced by broader culture c. *Beliefs*—health beliefs/behaviors influenced by belief system (e.g., religion)	*4a*. “The kids reiterate whatever the parents say at home. They're really not educating themselves, so educating the parents would help us educate the students...”—**Middle School Staff** *4b*. ‘My parents are from the Caribbean, so, yeah, so basically they have the [tea]…No just to… No, not to treat—like to prevent, yeah.'—**High School A Student** *4c*. "There was some people came to my church and said, ‘No, not a member of my church,' uh, ‘this is the mark of the beast.'—**High School B Staff**
5. Compatibility of Policies and Places with Healthy Behaviors	Contextual factors (e.g., the physical environment or policy structure) surrounding the individual that influence the ability to engage in health behaviors	*5a*. “Some school districts don't even have policies, you know. Some, a kid is in a class, he tested positive, and went to school the next day. Now there's three more cases in the class because they don't have a COVID policy.”—**Middle School Teacher**
6. Reliability of information	Concerns related to the trustworthiness of publicly available information about COVID-19. This includes: a) *Questionable information—*false, questionable or lack of information that leads to lack of confidence b) *Mixed messages*—information, rules, or behaviors that differ over time or vary by source or location c) *Politicized information*—information that is interpreted as political	*6a*. “Here's um, and not relying on this false information from social media from somebody who's rogue on YouTube and all they do is post videos all the time. Um, and that's the issue. And I think that people who can understand that can make an informed decision.”—**High School B Staff** *6b*. “A lot of the information that the CDC puts out, um, it's like they say one thing, and then within a few days or a few weeks, “Oh, don't, don't listen to that,” it's something different. And that's what has caused my apprehension with getting vaccinated.”—**Middle School Caregiver** *6c*. “One of the things in my family, I kept reminding them it is a health problem, not a political problem...that is why we stopped watching the news.”—**High School B Caregiver**
	d) *Mistrust—*feeling that information cannot be trusted due to general concerns about science, medical information, or sources of information and/or historical concerns related to historical medical and research abuse, particularly in the Black community	*6d*. “I think that with the African American population, um, we've been so, like, abused by the healthcare system that there's just a overall mistrust.”—**High School B Staff**
7. Perceived Risk	The spectrum of individuals' level of concern about the health impacts of COVID-19, including: a) *Apathy—*lack of concern about health impact of COVID-19 b) *Concern impacting thoughts—*fears about health of self, family, or community c) *Concern impacting behaviors*– engaging in or ignoring recommended health behaviors due to perceived levels of safety	*7a*. “If I put a number on it, I would say 99% of the students don't have a concern at all. There might be 1% that do have a concern. My perspective with the parents: They're over it.”—**High School B Staff** *7b*. [when asked about COVID-19 concerns] “Um, people that are dying, ‘cause I just had a recent relative that passed away from that.”—**High School A Student** *7c*. “I took my booster, um, [date]. I still protect myself, because you never know who you're around. You have to.”—**High School A Staff**

**Table 3:** Frequency of Individuals endorsing themes by role.

**Theme/subtheme**	**Student *n* (%)**	**Staff *n* (%)**	**Caregiver *n* (%)**	**Total *n* (%)**
**1**. Vaccine hesitancy	4 (44.4%)	18 (69.2%)	13 (76.5%)	35 (67.3%)
1a. Potential side effects	4 (44.4%)	13 (50.0%)	6 (35.3%)	23 (44.2%)
1b. Vaccine development	1 (11.1%)	4 (15.4%)	4 (23.5%)	9 (17.3%)
1c. Content of vaccine	2 (22.2%)	4 (15.4%)	3 (17.6%)	9 (17.3%)
1d. Vaccine effectiveness	0 (0.0%)	12 (46.2%)	7 (41.2%)	19 (36.5%)
1e. Age of recipient	1 (11.1%)	5 (19.2%)	4 (23.5%)	10 (19.2%)
**2**. Testing hesitancy	2 (22.2%)	5 (19.2%)	5 (29.4%)	12 (23.1%)
2a. Reliability of testing	1 (11.1%)	3 (11.5%)	3 (17.6%)	7 (13.5%)
2b. Consequences of positive test	1 (11.1%)	2 (7.7%)	1 (5.9%)	4 (7.7%)
**3**. Developmental stage	1 (11.1%)	5 (19.2%)	3 (17.6%)	9 (17.3%)
**4**. Cultural and family traditions and beliefs	1 (11.1%)	13 (50.0%)	6 (35.3%)	20 (38.4%)
4a. Family practices	0 (0.0%)	5 (19.2%)	1 (5.9%)	6 (11.5%)
4b. Cultural practices	1 (11.1%)	3 (11.5%)	1 (5.9%)	5 (9.6%)
4c. Beliefs	0 (0.0%)	12 (46.2%)	5 (29.4%)	17 (32.7%)
**5**. Compatibility of policies and places with recommended health behaviors	4 (44.4%)	9 (34.6%)	5 (29.4%)	18 (34.6%)
**6**. Reliability of information	2 (22.2%)	21 (80.8%)	12 (70.6%)	35 (67.3%)
6a. Questionable information	0 (0.0%)	7 (26.9%)	7 (41.2%)	14 (26.9%)
6b. Mixed messages	2 (22.2%)	11 (42.3%)	10 (58.8%)	23 (44.2%)
6c. Politicized information	1 (11.1%)	14 (53.8%)	4 (23.5%)	19 (36.5%)
6d. Mistrust	0 (0.0%)	8 (30.8%)	3 (17.6%)	11 (21.2%)
**7**. Perceived risk	6 (66.6%)	16 (61.5%)	12 (70.6%)	34 (65.4%)
7a. Apathy	1 (1.1%)	7 (26.9%)	2 (11.8%)	10 (19.2%)
7b. Concern impacting thoughts	2 (22.2%)	10 (38.5%)	8 (47.1%)	20 (38.5%)
7c. Concern impacting behaviors	5 (55.5%)	9 (34.6%)	10 (58.8%)	24 (46.2%)
Total participants per role	9	26	17	52

Note: The Staff category includes one participant who identified as both staff and caregiver; the theme may have been referenced by more participants than the sub-themes, because some excerpts were placed in an “other” sub-theme that did not ultimately constitute a pattern warranting its own sub-theme.

### Theme 1: Vaccine hesitancy

Participants expressed significant hesitations regarding the COVID-19 vaccine. Specifically, participants were concerned about *potential side-effects, vaccine development*, the *content of vaccines, vaccine effectiveness*, and *age of recipient*. Concerns about potential side effects of the vaccine were most frequently cited, followed by concerns about its effectiveness. Concerns about vaccine effectiveness were described by caregivers and staff only. *Side effects* of concern included death (1a), allergic reactions, infertility, and less frequently, vaccines turning individuals into zombies or turning the injection site magnetic. Participants cited concerns regarding *vaccine development* including the unknown long-term effects of the vaccine (1b) and the speed in which vaccines were developed. The safety of vaccine *contents* was also debated, for example, questioning if some unknown contents such as allergens (1c) or animal DNA were present. Concerns about *vaccine effectiveness* were often related to mixed messages from the media or health agencies. For example, participants described confusion about initial reports that vaccines would prevent illness, followed later by reports that vaccinated individuals could still contract COVID-19 (1d). Participants also described worry about becoming ill even if vaccinated. Finally, several participants reported concerns directly related to the *age of vaccine recipients*, especially among caregivers more hesitant to vaccinate younger children relative to older children or adults (1e).

### Theme 2: Testing hesitancy

Hesitations about testing were cited far less frequently than those surrounding vaccination. However, participants reported concerns about COVID-19 testing procedures and consequences related to subsequent test results. Specific concerns about the *reliability* of test results, included potential for false positives (2a) and inconsistent test results from multiple tests. Concerns regarding *consequences* of testing positive for COVD-19 (2b), including disruptions in work and social activities, were described as especially relevant for students due to school quarantine protocols at the time.

### Theme 3: Developmental stage

Developmental stage was perceived as a potential factor in ability to engage in consistent, effective health behaviors that would mitigate COVID-19 (3a). This was most often aired as a concern by caregivers and school staff. For example, children were perceived as being unable to engage effectively in certain health behaviors such as keeping their hands to themselves and wearing masks, due to age-typical behavioral tendencies (e.g., frequent hugging, sharing drinks).

### Theme 4: Cultural and family traditions and beliefs

Traditions and beliefs on multiple levels (i.e., familial, cultural) were reported to impact health decisions, especially vaccination or treatment approaches. Often, health decisions were made consistently for an entire family, influenced by their family values (4a). On a broader cultural level, some cultural traditions, like drinking a medicinal tea, were described as preventative or treatment approaches (4b). Beliefs, such as religion, also influenced decision-making (4c). These considerations were mentioned across schools and participant types, though they were most often discussed by staff, who recounted personal stories from their communities as well as anecdotes they had been told by students.

### Theme 5: Compatibility of policies and places with recommended health behaviors

The influence of contextual factors such as policies or physical environments influenced individuals' ability to engage in health behaviors. Caregivers described difficulties with socially distancing from family members living in the same home, and across participants the challenges with socially distancing in crowded school settings and abiding by quarantine recommendations when schools have lax COVID-related policies were noted (5a).

### Theme 6: Reliability of information

Participants expressed concerns regarding the trustworthiness of publicly available information about COVID-19. Specifically, participants perceived information shared on the news or on social media as *questionable* (6a). *Mixed messages* from official agencies such as frequently changing information throughout the pandemic from the CDC (6b) and conflicting recommendations across agencies (e.g., different messages from CDC vs. local or state officials) further contributed to the lack of trust in COVID-related information. Participants also expressed unease about the *political* nature of COVID rhetoric, which in some cases led to ignoring COVID-19 news altogether (6c). Finally, general *mistrust* in science, medicine, or other sources of information and specific mistrust related to historical medical abuse of Black populations (6d) were reported to negatively influence the trustworthiness of publicly available COVID-19 information. Overall, students raised far fewer concerns about reliability of information than caregivers or school staff.

### Theme 7: Perceived risk

Participants expressed a range of concern regarding the impact of COVID-19 on their health and/or the health of their broader family or community. The majority of students and families exhibited *apathy* or lack of concern about the health impacts of the COVID-19 virus (7a). Others expressed *concern impacting their thoughts*, or worry, about illness and death resulting from COVID-19, especially those who experienced the death of a loved one (7b). Participants provided anecdotes of these fears among staff, caregivers, and students. Sometimes these *concerns were directly linked to decisions about health behaviors*. For example, several participants described that concern for their health motivated them to engage in health behaviors like vaccination (7c). By contrast, others reported that people do not engage in health behaviors because they feel safe or protected from it for reasons such as being vaccinated, believing COVID-19 is like a cold, or feeling safe around their family members.

## Discussion

Using qualitative methods, this study examined factors associated with COVID-19 perceptions and health decisions among key stakeholders in racially and ethnically diverse school communities. Participants identified several factors influencing their health decisions in response to COVID-19. These factors spanned multiple complex ecological systems, including perceived individual risk, family values, media messaging, and cultural practices.

Factors related to testing and vaccine hesitancy in our sample were generally consistent with findings from other studies. For example, a scoping review of testing hesitancy also identified consequences of testing among the common barriers to testing in five other studies, though concerns about reliability of tests were not reported (41). Similar to other qualitative studies of diverse populations [e.g., (42–44)], numerous participants who expressed hesitation about the COVID-19 vaccine reported concerns about its safety and effectiveness. Many felt that the vaccine was developed too fast and that its long-term effects are not fully understood. Notably, in this sample, the scarcity of research on the vaccine in younger children served as a significant barrier for some caregivers regardless of their own vaccination status. For context, the United States Food and Drug Administration had not approved a COVID-19 vaccine for children under 5 years old at the time of data collection and approved the vaccine for children ages 5 through 11 during the data collection period in October, 2021. Concerns about vaccine decision making were often linked to consistency of and trust in available health information.

Frequently changing and conflicting recommendations related to COVID-19 contributed to feelings of mistrust and perceived misinformation from public health officials among participants. Participants wanted better justification for changes in guidelines. They also acknowledged the powerful influence of anecdotal narratives about the COVID-19 vaccine, even if these conflicted with advice from public health agencies. The proliferation of misinformation about vaccines and infectious disease has been present for years, particularly given the influence of social media (45). This is especially concerning for youth, who spend significant time on social media platforms, yet in our sample were less likely to raise concerns about reliability of information than adults. Challenges with eHealth literacy (i.e., the ability to seek, find, understand, and appraise information from eHealth resources such as scientific and health information via online media) may have significant health consequences, such as delayed help seeking (46). These findings underscore the importance of clear and accurate language in public health messaging, greater transparency about what is known and what is not known about COVID-19 and the vaccine, the need for positive narratives about vaccination from trusted sources, and a potential role for health professionals for filtering and disseminating trustworthy information.

Given the opportunity for health professionals take on the role of purveyors of trustworthy information, historically rooted mistrust in the healthcare system must be acknowledged. Historical and structural racism and discrimination were identified by participants as associated with mistrust in health information and engagement in health behaviors. Several participants referenced the Tuskegee syphilis study as an example of government-sanctioned medical maltreatment of marginalized communities. Experiences of perceived discrimination among communities of color continue to persist today. A recent study found that one in six Black individuals reported negative experiences with health care professionals (47). Therefore, it is important that outreach programs targeting racial and ethnic minority communities address these perceived injustices and work to build trust.

When working with school communities, there are additional opportunities for intervention at the family, school, and community levels. Participants highlighted the influence of family values, belief systems, and school policies on their health beliefs and behaviors. A comprehensive intervention to improve health of school-aged children may therefore involve any or all of these influences, for example through parent-level intervention, targeting faith communities, and addressing state or local policies on mitigation strategies such as social distancing, masking, and/or vaccination.

Perceptions of risk for COVID-19 infection or illness varied in our sample. COVID-19 risk perception has been found to have a direct effect on protective and preventative health behaviors such as distancing, hand hygiene, and mask wearing (48). In the present study, many adults reported taking action to prevent the spread of COVID-19 (e.g., masking, vaccination) for either their personal health and/or the health of others. These actions are consistent with a national study of over 1,000 parents in which Hispanic and Black parents reported being four times more likely to have kept their child out of school due to COVID-19 concerns compared to White parents in January, 2022 (49). However, participants in this sample reported perceptions that youth were unable to understand the risks or control their behaviors due to developmentally typical behaviors (e.g., frequent hugging, sharing drinks) and therefore were not engaging in the recommended risk mitigating behaviors. Future studies should further examine parental decision-making and motivating factors for adolescents to identify the best strategies to protect school-aged youth from COVID-19.

Of note, concerns related to testing and vaccine access or immigration status did not emerge in the current study despite being noted in other qualitative and quantitative studies about COVID-19 with similarly diverse communities (43, 50–52). This implies that barriers to vaccination and testing may differ across communities. Additionally, the primary barriers to vaccination or health behaviors defined by researchers may not align with the priorities of communities. By using methods of CBPR, which focuses on priorities of community members (26) and qualitative research, which centers the voices of participants (29) researchers can ensure that community needs are at the forefront of research and intervention efforts.

## Limitations

This study has several limitations. First, the distribution of caregiver, staff, and student participants were uneven such that adult perspectives are more represented in our study. However, 34.6% of adult participants identified as being a caregiver and caregivers ultimately make medical decisions for their children. Applying an inductive approach to the analysis allowed us to capture unique themes within each focus group and by participant type (e.g., student vs. caregiver) to examine differences and similarities in perspectives across participants. However, we did not collect data to identify the specific child/caregiver dyads and were therefore unable to examine these relationships in further detail. It is possible that including students and their caregivers in the same focus groups may have impacted their comfort expressing opinions due to expectations within the student or caregiver role. All participants were encouraged to respond to questions, but given the group format of focus groups, it is possible that certain participants may dominate the conversation. Our sample may have experienced more concerns, investment, and/or impact about COVID than the general school community, due to self-selection bias. The sample might have been more invested or affected by COVID-19 and as a result self-selected to participate in the study. Lastly, these results represent a snapshot in time. Focus groups were conducted during the fall of 2021, when testing was widely available in the area where data were collected, and vaccines were approved for children ages 12 and older. At the end of October 2021, after some, but not all, of those focus groups were conducted the first COVID-19 vaccine was approved for children ages 5 through 11. Concerns may evolve as knowledge about the virus evolves and as vaccines are made available to larger groups of individuals.

## Conclusions and future directions

Racial and ethnic minority communities have been disproportionately affected by COVID-19, but were slow to engage in infection mitigating behaviors, such as vaccination and testing. In this study, participants from a primarily racial and ethnic minority school community identified factors contributing to their perceived risk and engagement in COVID-19 mitigating behaviors that are described in detail in this paper. These data have the potential to inform tailored health promotion interventions that may reduce COVID-19 health disparities for this and similar communities. Drawing on the CBPR framework, the study team intends to incorporate these stakeholder perspectives in the development of a pilot health promotion educational intervention to improve healthy behaviors and attendance at schools in the face of COVID-19. Furthermore, the knowledge gained in this study could inform interventions to promote uptake of health behaviors like vaccination and testing for other infectious diseases and/or in future pandemics.

## Data availability statement

The raw data supporting the conclusions of this article will be made available by the authors, without undue reservation.

## Ethics statement

The studies involving human participants were reviewed and approved by University of Miami IRB. Written informed consent to participate in this study was provided by the participants' (if aged 18 or older) or by a parent/legal guardian (if aged 17 or younger).

## Author contributions

VH, LG, and EP contributed to the conception and design of the study. TK, SH, AD, HZ, and NA contributed to the qualitative analysis. CN and KS performed the quantitative analysis. TK wrote the first draft of the manuscript. SH, AD, HZ, JM, KS, and EP wrote sections of the manuscript. All authors contributed to manuscript revision, read, and approved the submitted version.

## Funding

Research reported in this paper was fully supported by the Eunice Kennedy Shriver National Institute of Child Health and Human Development (NICHD) of the National Institutes of Health under agreement number OT2HD108111. The content is solely the responsibility of the authors and does not necessarily represent the views of the National Institutes of Health.

## Conflict of interest

The authors declare that the research was conducted in the absence of any commercial or financial relationships that could be construed as a potential conflict of interest.

## Publisher's note

All claims expressed in this article are solely those of the authors and do not necessarily represent those of their affiliated organizations, or those of the publisher, the editors and the reviewers. Any product that may be evaluated in this article, or claim that may be made by its manufacturer, is not guaranteed or endorsed by the publisher.
