# Supplementary material for: Community voices on factors influencing COVID-19 concerns and health decisions among racial and ethnic minorities in the school setting
Source: Front Public Health. 2022 Oct 19;10:1002209. doi: 10.3389/fpubh.2022.1002209 (PMC9627500; doi:10.3389/fpubh.2022.1002209)
Supplement: Supplementary file 1 [file Data_Sheet_1.PDF]

Supplemental Material

Guiding questions about COVID-19 concerns for focus groups

1. Current knowledge about Covid-19
    a. What are the current concerns about COVID, if any?
        i. have you heard among **students** about Covid-19?
        ii. [For adults] What about **yourself or [other] school staff**?
    b. Are there any cultural or family traditions (e.g., race/ethnicity, religion, cultural beliefs about disease and treatment) that you feel influence
        i. how **students** think about the Covid-19 disease, COVID-19 testing, or vaccination?
        ii. [For adults] What about **yourself or [other] school staff**?
    c. Some people seek information or treatment from different types of sources, such as doctors, helpers or healers.
        i. Where do you think most **students** seek information about and support for Covid-19 related topics (e.g., disease, testing, vaccination)?
        ii. [For adults] What about **yourself or [other] school staff**?

2. Hesitations about vaccination
    a. Are you aware of any specific thoughts or beliefs about the COVID-19 vaccine among **students** (e.g., it's development, efficiency, side effects)?
    b. Are you aware of any **students** who have you been vaccinated for Covid-19?
        i. If so, what reasons did they provide for getting vaccinated (e.g., concerns for health, concerns for public health, influential person's advice, convenience, etc.)?
        ii. If not, what are some things that prevented them from getting vaccinated (e.g., parents, self or family beliefs, money, work or family commitments, discrimination, didn't know where to get help, lack of services that understand language or background)?
    c. Are you aware of any specific thoughts or beliefs about the COVID-19 vaccine among **school staff** (e.g., it's development, efficiency, side effects)?
        i. What are motivators for vaccination for **yourself or school staff** (e.g., concerns for health, concerns for public health, influential person's advice, convenience, etc.)?
        ii. What are some things that may prevent **school staff** from getting vaccinated (e.g., beliefs, money, work or family commitments, discrimination, didn't know where to get help, lack of services that understand language or background)?

3. Hesitations about testing (*if time permits*)
    a. Are you aware of any specific thoughts or beliefs about COVID-19 testing among **students**?
    b. Are you aware of any **students** who have you been tested for Covid-19?

        i. If so, what reasons did they provide for getting tested (e.g., concerns for health, concerns for public health, exposure, referred/recommended, availability)?
        ii. If not, what are some things that prevented them from getting tested (e.g., parents, self or family beliefs, work or family commitments, discrimination, didn't know where to get help, lack of services that understand language or background)?
    c. Are you aware of any specific thoughts or beliefs about COVID-19 testing among **school staff**?
        i. What are motivators for vaccination for **yourself or school staff** (e.g., concerns for health, concerns for public health, exposure, referred/recommended, availability)?
        ii. What are some things that may prevent **school staff** from getting vaccinated (e.g., beliefs, inconvenience, work or family commitments, discrimination, didn't know where to get help, lack of services that understand language or background)?
    d. What are your expectations of Covid-19 testing?
        i. What are your past experiences with testing?

4. Is there anything we did not ask that you would like to discuss regarding Covid testing, vaccination and education?
